# Silicon Carbide Nanowire Based Integrated Electrode for High Temperature Supercapacitors

**DOI:** 10.3390/ma17164161

**Published:** 2024-08-22

**Authors:** Shiyu Sha, Chang Liang, Songyang Lv, Lin Xu, Defu Sun, Jiayue Yang, Lei Zhang, Shouzhi Wang

**Affiliations:** 1 School of Energy and Power Engineering, Shandong University, Jinan 250100, Chinajy_yang@sdu.edu.cn (J.Y.); 2 State Key Laboratory of Crystal Materials, Institute of Crystal Materials, Shandong University, Jinan 250100, China

**Keywords:** SiC crystal, integrate electrode, high temperature supercapacitors

## Abstract

Silicon carbide (SiC) single crystals have great prospects for high-temperature energy storage due to their robust structural stability, ultrahigh power output, and superior temperature stability. However, energy density is an essential challenge for SiC-based devices. Herein, a facile two-step strategy is proposed for the large-scale synthesis of a unique architecture of SiC nanowires incorporating MnO_2_ for enhanced supercapacitors (SCs), arising from the synergy effect between the SiC nanowires as a highly conductive skeleton and the MnO_2_ with numerous active sites. The SiC@MnO_2_ integrated electrode-based SCs with ionic liquid (IL) electrolytes were assembled and delivered outstanding energy and power density, as well as a great lifespan at 150 °C. This impressive work offers a novel avenue for the practical application of SiC-based electrochemical energy storage devices with high energy density under high temperatures.

## 1. Introduction

Supercapacitors (SCs) are one of the energy-related technologies developed in recent years that are working toward carbon neutrality, thanks to their eco-friendly, safe, and ultralong service lifespan [1]. Although tremendous progress has been made in developing supercapacitors, their energy density indicators are still struggling to meet commercial standards compared to lithium-ion batteries (LIBs) [2,3]. In SC systems, the electrode material is the most crucial element in determining the performance of the energy storage device. Up until now, SCs could be divided into two major types of electronic double-layered capacitors (EDLCs) and pseudocapacitors based on different energy storage mechanisms [4]. EDLC materials (such as carbon materials) are known to be the most promising candidates, but they suffer from unsatisfactory capacitance performance [5]. The pseudocapacitive electrode has attracted extensive attention due to its excellent charge storage properties originating from reversible redox reactions [6,7]. To enhance the energy capacity of devices, it is beneficial to cleverly merge the EDLC electrode with a pseudocapacitive material that offers both quick power output and high energy density. This approach has significant potential to overcome the constraints of each component and leverage their strengths effectively [8,9].

Silicon carbide (SiC), as a representative of wide-bandgap semiconductors, has widespread applications in optoelectronic devices [10,11,12,13,14], particularly under high-temperature conditions because of its advantages of excellent electron mobility, low thermal expansion, and superior stability [14,15,16]. The working temperature has a significant impact on the electrochemical performance of supercapacitors, especially in extreme environments. It is crucial to fundamentally understand the effects of temperature on capacitance and cycle life [15]. For instance, Li et al. reported a 4H-SiC nanochannel array electrode with an areal capacitance of 14.8 mF cm^−2^ at 10 mV s^−1^, and it reached a 96.8% retention of initial capacitance after 11,000 cycles at 60 °C [17]. Li et al. successfully prepared SiC nanowires on carbon fabric substrates, and the devices had an areal capacitance of 18.5 mF cm^−2^ at 2 mA cm^−2^ and a capacitance retention of 80% at the high temperature of 150 °C [18]. However, the areal capacitance of the most commonly reported pure SiC electrodes rarely exceeded those of today’s LIBs. In the past few years, researchers have devoted considerable effort to improving the energy density of SiC-based SCs [19].

Transition metal oxides/hydroxides such as MnO_2_ have been considered an appropriate substance for high-performance SCs because of their ideal theoretical capacity (1370 F g^−1^), multi-electron redox behaviors, abundance in the earth, and cost-effectiveness [20]. For instance, Pan and colleagues synthesized an MnO_2_ material on a carbon cloth base using the hydrothermal technique, achieving a specific capacitance of 3.22 F cm^−2^ [21]. Wang and colleagues created a hollow core–shell MnO_2_ heterostructure film using the interface-modified Kirkendall process, achieving an impressive areal capacitance of 4762 mF cm^−2^ despite a high mass loading of 30 mg cm^−2^ [22]. Based on the research mentioned, a SiC crystal with vertical nanochannels serving as a skeleton structure and MnO_2_ as the active material has been developed to create high-performance SiC-based supercapacitors. The SiC crystal acts as a sophisticated conductive base, reducing the transfer distance for ions/electrons to enhance reaction kinetics and providing numerous sites for the uniform deposition of MnO_2_ active materials, thereby enhancing overall electrochemical performance.

Herein, the porous SiC@MnO_2_ nanoarray is fabricated through simple and effective electrochemical etching and hydrothermal treatment technologies. The silicon carbide (SiC) supercapacitors are created using a SiC@MnO_2_ nanoarray for the electrodes paired with ionic liquid (IL) as the electrolyte, resulting in effective electrochemical performance at a temperature of 150 °C. On one side, the SiC single-crystal nanoarray provides enough space to enhance the stability of the electrode structure, while the MnO_2_ active material with a high theoretical capacity offers plenty of active sites to improve the adsorption/desorption of electrolyte ions. Conversely, ionic liquids (ILs) with broad voltage ranges and thermal stability have been gaining attention in research as electrolytes for high-temperature energy storage devices with satisfactory power density [23]. As a result, silicon carbide (SiC) supercapacitors have an energy density of 362 mWh cm^−2^ at 150 °C. This study offers important insights for achieving high-energy density SiC electrodes.

## 2. Experiment Section

**Porous N-type SiC nanoarrays:** An N-type SiC single-crystal material with a penetrating microporous structure was prepared using the PVT method. Highly ordered porous SiC nanoarrays were prepared using a modified two-step electrochemical anodization technique. In the first step, electrochemical etching experiments were carried out on N-type SiC single-crystal wafers in a configured saturated NF_4_HF_2_ solution using an applied power supply of 18 V for a set time of 10 min, with the main objective of removing the superficial layer structure from the wafer surface. In the second step, the wafers were etched in a 50 mL solution configured with anhydrous ethanol, HF, and H_2_O_2_.

**SiC@MnO_2_ nanocomposite:** MnO_2_ was deposited on the surface of the porous N-type SiC single-crystal electrode by the hydrothermal method, then 20 mL of 0.03 mol L^−1^ KMnO_4_ solution was configured, and the prepared porous electrodes were placed in a 50 mL polytetrafluoroethylene reactor. The experimental conditions were 160 °C for 3 h. After removal, the surface of the electrode material was rinsed with deionized water and dried in a vacuum-drying oven at 70 °C to obtain the SiC@MnO_2_ composite electrode.

**Three-electrode system and symmetric supercapacitor device preparation and characterization:** A SiC@MnO_2_ composite material was directly used as the electrode, a Ag/AgCl electrode was used as the reference electrode, a platinum sheet electrode was used as the counter electrode, and 2 M H_2_SO_4_ was used as the electrolyte. The voltage window for CV curve testing was −0.8 to −0.1 V, and the voltage window for GCD charge–discharge curve testing was also −0.8 to −0.1 V. The SiC@MnO_2_ composite material was assembled into a symmetric supercapacitor, with EMImNTf_2_ as the electrolyte and a voltage window of 0 to 1 V. The electrode was placed in an Ar oven and connected to external testing instruments via copper wires for high-temperature electrochemical experiments. In two-electrode measurements, the CV and GCD test data were collected in a temperature range from 25 °C to 150 °C using a special high-temperature explosion-proof box. Characterization methods such as SEM, XRD, and XPS were used to analyze the morphology and elemental composition of the electrode materials.

## 3. Results and Discussion

**Figure 1:**
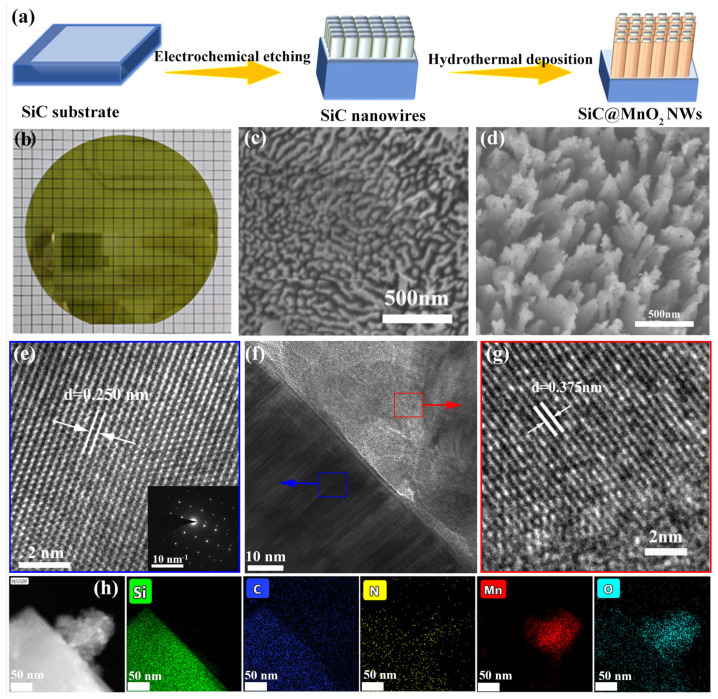
The schematic diagram of the synthesis process of the SiC@MnO_2_ composite material (**a**); the SEM images of N-type SiC (**b**), the SiC NWs (**c**), and SiC@MnO_2_ material (**d**). The TEM (**f**) and HRTEM image (**e**,**g**) of the SiC@MnO_2_ and corresponding SAED pattern ((**e**) inset); (**h**) HADDF-TEM image and element (Si, C, N, Mn and O) mapping images of SiC@MnO_2_, respectively.

The process of creating a SiC@MnO_2_ nanoarray is illustrated in Figure 1a, showing a SiC single-crystal substrate and MnO_2_ nanosheets grown on the surface of the SiC nanoarray. Firstly, N-type SiC single crystals (Figure 1b) were selected to form a SiC nanoarray via anodic oxidation technology. Then, the MnO_2_ active material was anchored onto the SiC nanoarray surface via hydrothermal reaction and annealing treatment. It was observed that the SiC wafers changed from a light green color to a dark green color after MnO_2_ deposition, signifying the generation of MnO_2_ active material on the SiC surface.

The morphologies and microstructures of the as-synthesized samples were examined through a scanning electron microscope (SEM). Vertically aligned nanoarrays with a columnar-like appearance were homogeneously distributed in the SiC wafer surface (Figure 1c). From the SEM analysis, the diameter of the SiC nanoarrays is approximately 20 nm, which provides enough space for electrolyte–electrode contact to enhance electrolyte ion accessibility and diffusion capability. Moreover, it can be found that the SiC nanoarray surface becomes rough, suggesting manganese-based precursor components uniformly cover the SiC nanoarray surface with a superior mechanical connection. The carefully crafted microstructures prevent the clumping together of Mn-based active material, allowing for more active sites to be exposed and enhancing capacitive storage. Following calcination, the precursor components of manganese are transformed into MnO_2_ due to the evaporation of water, leading to the creation of a SiC@MnO_2_ nanoarray. 

The transmission electron microscope (TEM) further confirms the microscopic morphology and structural evolution of the SiC@MnO_2_ nanoarray. The low-magnification TEM image (Figure 1f) shows discernible interfaces between the SiC nanoarray and MnO_2_ nanoparticle, providing powerful evidence that MnO_2_ active material with a size of several nanometers is successfully anchored onto the SiC nanoarray surface. It is worth noting that the MnO_2_ nanoparticle presents an ultrathin architecture, which contributes to a larger specific surface area for energy storage. The high-resolution TEM image (Figure 1e,g) verifies two different lattice spacings of 0.250 nm and 0.375 nm, which are well matched to the (004) crystal planes of SiC single crystals and (003) crystal planes of MnO_2_ [24]. In the selected area electron diffraction (SAED) pattern of SiC@MnO_2_ (Figure 1e inset), there is a set of diffraction spots representing the single-crystal structure of the obtained SiC nanoarray and the polycrystalline structure of MnO_2_. The selected area element mapping identifies the homogeneous distribution of Si, C, N, O, and Mn elements over the entire nanoarray.

**Figure 2:**
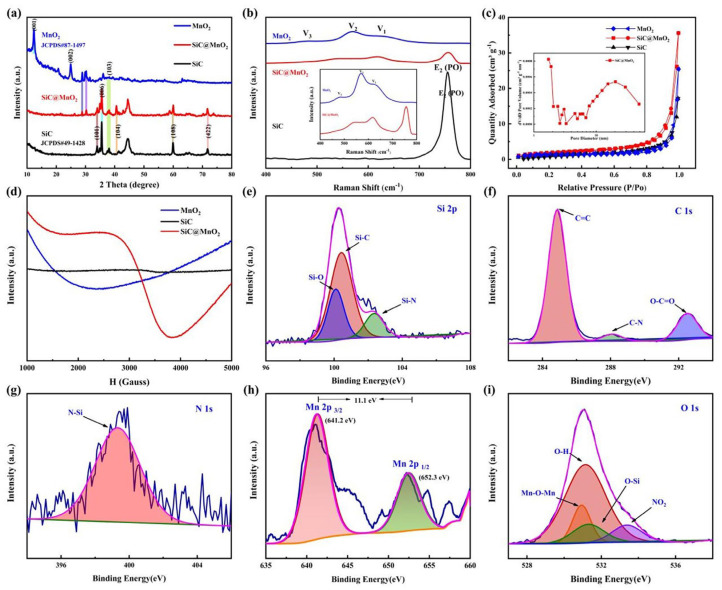
Structural characterization of SiC@MnO_2_−based materials. XRD pattern (**a**); Raman (**b**); N_2_ adsorption/desorption isotherms (**c**); EPR spectra (**d**); high−resolution XPS spectra for the Si 2p (**e**), C 1s (**f**), N 1s (**g**), Mn 2p (**h**), and O 1s (**i**).

To determine the phase compositions of as-prepared products, the X-ray diffraction (XRD) pattern is displayed in Figure 2a. It is found that all characterized peaks of the MnO_2_ composite are indexed to the SiC (JCPDS: 49–1428) and MnO_2_ (JCPDS: 87–1497), indicating that the MnO_2_ nanosheets are successfully introduced into the SiC nanocolumns without changing the hexagonal SiC crystal structure. In addition, no additional peaks are observed in the SiC@MnO_2_ composite, suggesting its purity. Similar findings can also be observed in the Raman spectra. As shown in Figure 2b, there are two significant Raman peaks located at 756.30 and 618.57 cm^−1^, which correspond well to the Mn-O stretching modes of the MnO_2_ and E_2_(PO) modes of SiC crystal [25,26,27], respectively, confirming the existence of two phases in the SiC@MnO_2_ samples, in agreement with the XRD result. In comparison with the SiC crystal and MnO_2_, the peak position of the SiC@MnO_2_ complexes is slightly shifted, which ascribes to the potential interaction between the SiC substrate and MnO_2_ active material.

By analyzing the nitrogen adsorption and desorption curves, along with the pore size distribution curves (Figure 2c and inset), it was observed that the SiC@MnO_2_ composite exhibits a characteristic III isotherm with an H3 hysteresis loop at elevated pressures. This suggests the presence of abundant mesoporous structural characteristics [28], which stem from the spaces between SiC@MnO_2_ nanoarrays. The SiC@MnO_2_ nanoarray exhibits a considerable increase in specific surface area of 6.86 m^2^ g^−1^ with a pore diameter of approximately 11 nm compared with SiC single crystals (4.17 m^2^ g^−1^) and MnO_2_ (4.13 m^2^ g^−1^). The numerous mesopores contribute to providing a fast transport channel for electrolyte ions.

The electron paramagnetic resonance (EPR) spectra (Figure 2d) reveal the sharp and symmetrical Dysonian peaks, which are ascribed to the absence of carbon and oxygen atoms in SiC single crystals and MnO_2_ nanoparticles. Significantly, the highest signal strength is observed in the SiC@MnO_2_ nanoarray, indicating an increase in vacancy concentration resulting from the merging of SiC single crystals and MnO_2_ nanoparticles [29]. These defects play a crucial role in facilitating electrolyte ion adsorption, enhancing electron transfer, and providing additional capacitive performance [30].

The surface elemental chemical valence of the SiC@MnO_2_ nanoarray is detected by utilizing X-ray photoelectron spectroscopy (XPS). In the detailed high-resolution (HR) Si 2p spectra (Figure 2e), the three distinct peaks observed at 100.09 eV, 100.42 eV, and 102.33 eV are associated with Si–O, Si–C, and Si–N bonds, respectively [31,32,33]. The presence of the Si-N bond is attributed to nitrogen doping in N-type SiC single crystals, which enhances the conductivity of the SiC substrate and correlates with the N 1s spectra fitting results (Figure 2g) [31,32,34]. The appearance of the Si-O bond further demonstrates that SiC single crystals successfully coordinated with the MnO_2_ active material, which aligns with XRD and Raman measures. In the C 1s spectra (Figure 2f), three peaks at 284.88 eV, 288.10 eV, and 292.52 eV were identified, corresponding to C=C, C–N, and O–C=O bonds, respectively [31,34,35,36]. As illustrated in Figure 2h, the fitted O 1s spectrum peaks at 530.96 eV, 531.15 eV, 531.34 eV, and 533.39 eV correspond to Mn–O–Mn, O–H, and Si–O bonds and surface-adsorbed oxygen species [37,38,39,40]. The Mn 2p spectrum consists of Mn 2p3/2 at 641.2 eV and Mn 2p1/2 peaks with a spin-orbit separation of 652.3 eV [25,34,41,42].

**Figure 3:**
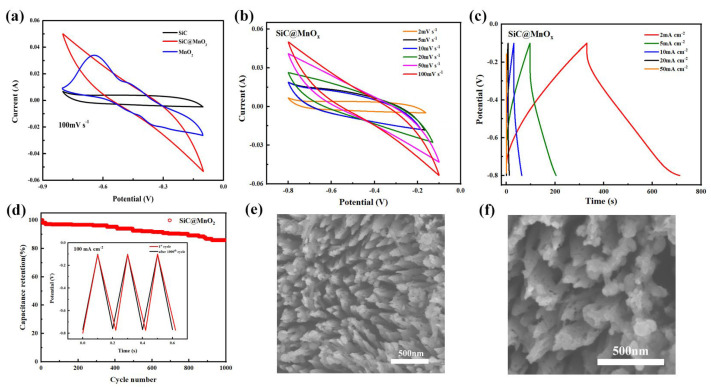
The three-electrode electrochemical performance of SiC@MnO_2_. The CV curves at 100 mV s^−1^ (**a**) and 2−100 mV s^−1^ (**b**); the GCD curves (**c**); the capacitance retention and coulombic efficiency (**d**); and the SEM images of the electrode initially (**e**) and after 1000 cycles (**f**).

To evaluate the electrochemical performance of SiC@MnO_2_, a three-electrode system was executed in a 2 M H_2_SO_4_ aqueous electrolyte. The cyclic voltammetry (CV) curves at a scan rate of 100 mV s^−1^ were collected and are shown in Figure 3a. The CV curves for SiC@MnO_2_ nanoarrays present a larger CV area compared to SiC and MnO_2_, confirming their enhanced capacitive performance in comparison to that of SiC single crystals [43]. In addition, the CV curves of the SiC@MnO_2_ nanoarray hold a nearly rectangular shape, indicating the typical pseudocapacitive charge storage [44]. Moreover, the peak currents of SiC@MnO_2_ electrodes increased linearly and almost no visible distortion occurred when the scan rates expanded from 2 to 100 mV s^−1^ (Figure 3b), demonstrating favorable reversibility. The galvanostatic charge/discharge (GCD) profiles at the current density of 2–50 mA cm^−2^ under an identical voltage window range (Figure 3b) exhibited linear and symmetrical behavior, suggesting excellent coulomb efficiency. The specific capacitance of the SiC@MnO_2_ electrode is determined based on the GCD curves. Benefiting from the perpendicular ion diffusion channels of the SiC substrate and the high theoretical capacity of the MnO_2_ active material, the SiC@MnO_2_ nanoarrays obtain the areal specific capacitance of 1034.28 mF cm^−2^ at 2 mA cm^−2^, 887.86 mF cm^−2^ at 5 mA cm^−2^, 705.71 mF cm^−2^ at 10 mA cm^−2^, and 250.71 mF cm^−2^ at 20 mA cm^−2^, showing acceptable rate capability. With the increasing current density and scan rates, the electrode also maintains relatively high specific capacity, which demonstrates the excellent rate capability of the SiC@MnO_2_ nanoarrays. More importantly, long-term cycling tests were carried out to justify the fast charging ability of the SiC@MnO_2_ electrode. As displayed in Figure 3d, the SiC@MnO_2_ complexes deliver 83.33% of the initial capacitance under the current of 100 mA cm^−2^ and the coulomb efficiency is close to 100% after 1000 cycles, signaling excellent structural stability. Undoubtedly, the strengthened cycling durability originates from the distinctive SiC nanoarray structure, which provides sufficient space to alleviate the volume expansion caused by the high current shock during the charging and discharging processes. The SEM images before (Figure 3e) and after (Figure 3f) 1000 cycles demonstrate that the morphology of the material does not show significant damage or changes, with only a slight reduction in the density of the porous arrays but still maintaining good structural integrity. Apart from some edges becoming blurred, the open ion channel structure is preserved. Our group has previously conducted other related work on MnO_2_ [45]. Manganese dioxide, as a metal oxide with high pseudocapacitance, can achieve high capacitance storage, but it has poor rate performance due to structural collapse during high current cycling. This is the main reason why we introduced N-type SiC with high structural stability and large specific surface area porosity.

**Figure 4:**
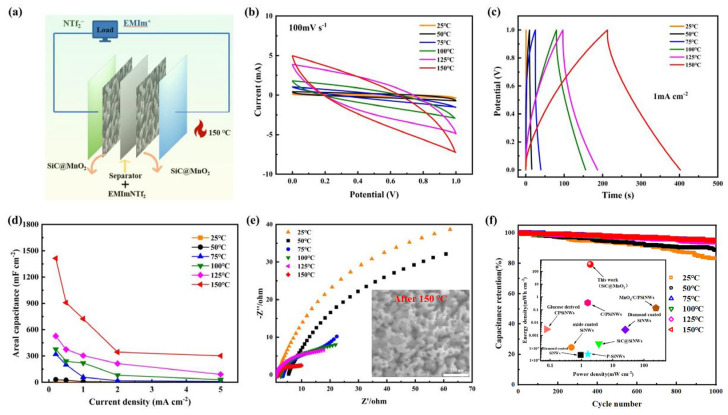
The electrochemical properties of SiC−based SCs in a high-temperature environment of 25–150 °C. (**a**) Schematic illustration of the devices; (**b**) CV curves at 100 mV s^−1^; (**c**) GCD curves at 1 mA cm^−2^; (**d**) rate performance; (**e**) EIS plots (the illustration is an SEM image after 150 °C); (**f**) the long−term cycling stability and the inset is a Ragone plot of the comparison with advanced high−temperature storage devices.

The SiC-based supercapacitors were constructed using a SiC@MnO_2_ nanoarray as the electrode and ionic liquids (ILs) as the electrolyte, as illustrated in Figure 4a. By leveraging the durability of SiC-based electrodes and the unique properties of ILs, this setup offers a blueprint for creating high-energy-density SiC-based supercapacitors with strong stability during high-temperature cycling. The CV curve of SiC-based SCs operating in different temperatures is shown in Figure 4b and the voltage range is determined to be 0−1 V after optimization to avoid high-temperature side reactions. At a scan rate of 100 mV s^−1^, the cyclic voltammetry (CV) curves all exhibit a rectangular shape. As the temperature increases from 25 to 150 °C, the enclosed area of the CV curve expands steadily without any significant distortion in shape. This indicates a high level of reversibility, and the temperature rise is beneficial for enhancing the capacitance performance. In Figure 4c, the GCD curve holds a quasi-triangular appearance under the temperature of 25–150 °C, and the longest discharge time is present at 150 °C, implying an improved electrochemical capacity, which is consistent with the CV conclusions. The areal capacitance of the SiC-based SCs is calculated and summarized in Figure 4d. At the high temperature of 150 °C, the SiC-based electrode achieves the areal capacitance of 302.0 mF cm^−2^ at 5 mA cm^−2^, which outperforms that of 25 °C. The improved charge storage properties are attributed to the enhancement of electrolyte ion mobility dynamics with elevated temperatures [46]. To deeply analyze the SiC-based electrode kinetics under different temperatures, an electrochemical impedance spectroscopy measure was conducted. From the typical Nyquist plot (Figure 4d), it can be found that the SiC@MnO_2_ electrode possesses the fastest ion diffusion capability at 150 °C. The intercepts with the X-axis and the diameter of the semicircle progressively decrease as the temperature rises from 25 to 150 °C, indicating a boosted affinity between the electrode and IL electrolyte, confirming the promoted mass/electronic transport kinetics and resulting in the ideal capacitive response at 150 °C [47,48]. The SiC-based SCs deliver 95% of the initial capacitance after 1000 cycles at the current density of 10 mA cm^−2^ when operated in a 150 °C atmosphere (Figure 4e). The ordered SiC nanoarray forms permeable channels for rapid ion adsorption and desorption, and the close bond between the SiC substrate and MnO_2_ is conducive to the structural stability of the SiC-based electrodes in the high-temperature environment. The SiC-based supercapacitors show a top areal power density of 1.905 mW cm^−2^ and a maximum energy density of 362 mWh cm^−2^ at 150 °C, surpassing the performance of many similar advanced devices documented in the existing literature (Table 1), such as diamond-coated Si NWs [49,50], SiC NWs on a SiC film [51], MnOX/C/Si NWs [52,53], C-Si NWs [54], and so on [55,56]. The Ragone plot is shown in Figure 4e.

## 4. Conclusions

In summary, SiC@MnO_2_ nanowires have been developed via the facile electrochemistry etching technique and the in situ growth method. Due to the synergy of the unique SiC nanowires’ conductive frameworks and the ideal theoretical capacity of MnO_2_, the SCs assembled with an SiC@MnO_2_ integrated electrode and IL electrolyte acquire satisfactory electrochemical performance at 150 °C. The SCs operate normally at a current density of 1 mA cm^−2^ at 150 °C, exhibiting power and energy densities of 1.905 mW cm^−2^ and 362 mWh cm^−2^, respectively. After 1000 cycles, the capacitance retention rate exceeds 95%. Therefore, based on the in-depth exploration of SiC and its composite properties, this study is expected to lead to the development of more high-performance and high-stability energy storage devices. This will not only broaden the application field of new semiconductor materials but also provide a reference for the development of energy storage and conversion methods.

## Figures and Tables

**Table 1:** Comparison of the electrochemical performance of advanced devices.

Electrode Materials	Energy Density	Power Density	Capacity Retained after Cycling	Reference
SiC@MnO_2_	362 mWh cm^−2^	1.905 mW cm^−2^	95% after 1000 cycles	This work
Diamond-coated Si NWs	84 μJ cm^−2^	0.94 mW cm^−2^	93.3% after 10,000 cycles	[49]
MnO_2_@ SiNWs	29.1 μWh cm^−2^	9.1 μWh cm^−2^	91% after 5000 cycles	[52]
3D Al@Ni@MnOx NSP	23.02 Wh kg^−1^	947.11 Wh kg^−1^	96.3% after 10,000 cycles	[53]
C@SiNWs	17.4 mJ m^−2^	0.351 W m^−2^	75% after 25,000 cycles	[54]
SiC nanowires	1.7 mJ cm^−2^	12 mW cm^−2^	90% after 100,000 cycles	[57]

## Data Availability

The original contributions presented in the study are included in the article, further inquiries can be directed to the corresponding author.
